# Elective Laparoscopic Transabdominal Preperitoneal Repair after Successful Manual Reduction of an Incarcerated Inguinal Hernia with Suspected Small-Bowel Ischemia: A Case Report

**DOI:** 10.70352/scrj.cr.26-0407

**Published:** 2026-10-24

**Authors:** Yoichi Miyaoka, Ryoji Yokoyama, Tomoya Saito, Daichi Hanaoka

**Affiliations:** 1 Department of Surgery, Abashiri-Kosei General Hospital, Abashiri, Hokkaido, Japan; 2 Postgraduate Clinical Training Program, Abashiri-Kosei General Hospital, Abashiri, Hokkaido, Japan

**Keywords:** incarcerated inguinal hernia, manual reduction, small-bowel ischemia, laparoscopic surgery, transabdominal preperitoneal repair (TAPP), case report

## Abstract

**INTRODUCTION:**

Incarcerated inguinal hernia is a common surgical emergency, and urgent surgery is generally required when bowel strangulation or ischemia is suspected. However, in carefully selected patients, successful manual reduction followed by close observation may allow repair to be performed electively during the same hospitalization. We report a case of incarcerated inguinal hernia with CT findings suggestive of impaired small-bowel perfusion that was successfully managed by manual reduction, inpatient observation, and same-admission elective laparoscopic transabdominal preperitoneal repair (TAPP).

**CASE PRESENTATION:**

A 78-year-old man was transferred to our hospital with painful right groin swelling that had become irreducible approximately 5 h before successful manual reduction. He had a history of an intermittent right groin bulge that had previously been self-reducible. On arrival, his vital signs were stable, and a right inguinal bulge was observed. Manual reduction was achieved shortly after examination, although the hernia recurred easily with increased abdominal pressure. Contrast-enhanced CT demonstrated a right inguinal hernia containing small bowel, with bowel wall thickening, mesenteric fat stranding, slightly reduced enhancement, and a small amount of ascites, suggesting possible bowel ischemia. Because his symptoms improved after reduction and there were no definite signs of peritonitis or bowel necrosis, he was admitted for close observation. After stabilization, elective laparoscopic TAPP was performed on hospital day 4. Intraoperatively, a segment of ileum showed reddish discoloration but had preserved peristalsis, and no necrosis or perforation was identified. A right indirect inguinal hernia was repaired with a 3D mesh using the transabdominal preperitoneal approach. The postoperative course was uneventful, and the patient was discharged without major complications. At 3 months after surgery, no recurrence, bowel obstruction, mesh-related complication, or chronic groin pain was observed.

**CONCLUSIONS:**

This case suggests that successful manual reduction followed by close inpatient observation may allow safe same-admission elective laparoscopic repair in carefully selected patients with incarcerated inguinal hernia, even when preoperative imaging suggests possible but not definitive bowel ischemia. The laparoscopic approach is useful not only for definitive hernia repair but also for direct assessment of bowel viability after reduction.

## Abbreviations

GPS
: Gentle, Prepared and Safe
TAPP
: transabdominal preperitoneal repair
WSES
: World Society of Emergency Surgery

## INTRODUCTION

Incarcerated inguinal hernia is a frequent emergency condition in general surgery. When bowel strangulation, necrosis, or perforation is suspected, urgent surgical intervention is generally required.^1,2)^ In contrast, successful manual reduction, sometimes referred to as taxis, may allow avoidance of immediate emergency surgery in selected patients, provided that there are no clinical signs of irreversible ischemia or peritonitis and that careful observation is performed after reduction.^3,4)^ The updated HerniaSurge guidelines suggest that manual reduction may be attempted in acutely irreducible groin hernias without suspicion of bowel ischemia, followed by a period of observation after successful reduction.^2)^

However, decision-making after successful reduction remains challenging. Although manual reduction can relieve incarceration, delayed bowel ischemia, perforation, persistent closed-loop obstruction, and reduction en masse remain important concerns.^3,4)^ The optimal timing of definitive hernia repair after reduction is also not fully established. Same-admission elective repair may be useful because it avoids prolonged waiting after an episode of incarceration while allowing surgery to be performed under more controlled conditions than emergency surgery.

Mesh repair is recommended as a first-choice technique for groin hernia repair, either by open or laparoendoscopic approaches, depending on patient factors and surgical expertise.^1,2)^ In emergency or potentially contaminated settings, mesh use requires individualized judgment based on bowel viability, the need for bowel resection, and the degree of contamination.^5)^ Laparoscopic TAPP has potential advantages in selected incarcerated hernias because it enables both inspection of the previously incarcerated bowel and definitive hernia repair.^6–9)^ Herein, we report a case of incarcerated right inguinal hernia with CT findings suggestive of impaired small-bowel perfusion that was successfully treated by manual reduction, close inpatient observation, and same-admission elective laparoscopic TAPP.

## CASE PRESENTATION

A 78-year-old man was transferred to our hospital because of painful swelling of the right groin. He had noticed an intermittent right groin bulge for approximately 1 year, which had usually been self-reducible. On the day of presentation, the bulge became irreducible and painful, and nausea developed. The interval from onset of incarceration to successful manual reduction was approximately 5 h. He was initially evaluated at another hospital and was transferred to our department with a suspected incarcerated right inguinal hernia.

His medical history included hypertension, benign prostatic hyperplasia, and hyperuricemia. He had self-discontinued regular treatment for hypertension. He had no known allergies and was not taking antithrombotic medication. He was independent in activities of daily living.

On arrival, he was alert and hemodynamically stable. His blood pressure was 146/82 mmHg, heart rate was 57 beats/min, oxygen saturation was 98% on room air, and body temperature was 36.6 °C. Physical examination revealed an egg-sized bulge in the right groin. Manual reduction was achieved shortly after examination; however, the hernia recurred easily with increased abdominal pressure.

Laboratory findings on admission and the following morning are summarized in **Table 1**. Laboratory tests on admission showed leukocytosis, with a white blood cell count of 13900/μL. The C-reactive protein level was 0.23 mg/dL, the D-dimer level was 5.4 μg/mL, the lactate dehydrogenase level was 227 U/L, and the creatine kinase level was 122 U/L. Contrast-enhanced CT demonstrated a right inguinal hernia containing small bowel. The incarcerated bowel and the oral-side small bowel showed wall thickening, mesenteric fat stranding, and slightly reduced enhancement, raising concern for impaired bowel perfusion. A small amount of ascites was also observed (**Fig. 1**).

**Table 1:** Laboratory findings on admission and the following morning

Variable	On admission	Following morning
White blood cell count (/μL)	13900	11600
Hemoglobin (g/dL)	14.9	13.7
Platelet count (×10^4^/μL)	22.7	22.9
Prothrombin time-international normalized ratio	1.09	Not measured
Activated partial thromboplastin time (s)	30.5	Not measured
D-dimer (μg/mL)	5.4	Not measured
Total protein (g/dL)	6.6	5.8
Albumin (g/dL)	3.8	Not measured
Total bilirubin (mg/dL)	0.7	0.9
Aspartate aminotransferase (U/L)	23	20
Alanine aminotransferase (U/L)	12	10
Lactate dehydrogenase (U/L)	227	206
Alkaline phosphatase (U/L)	78	64
Gamma-glutamyl transpeptidase (U/L)	17	13
Amylase (U/L)	67	49
Creatine kinase (U/L)	122	Not measured
Blood urea nitrogen (mg/dL)	17.1	18.8
Creatinine (mg/dL)	1.00	1.01
C-reactive protein (mg/dL)	0.23	2.07

**Fig. 1:**
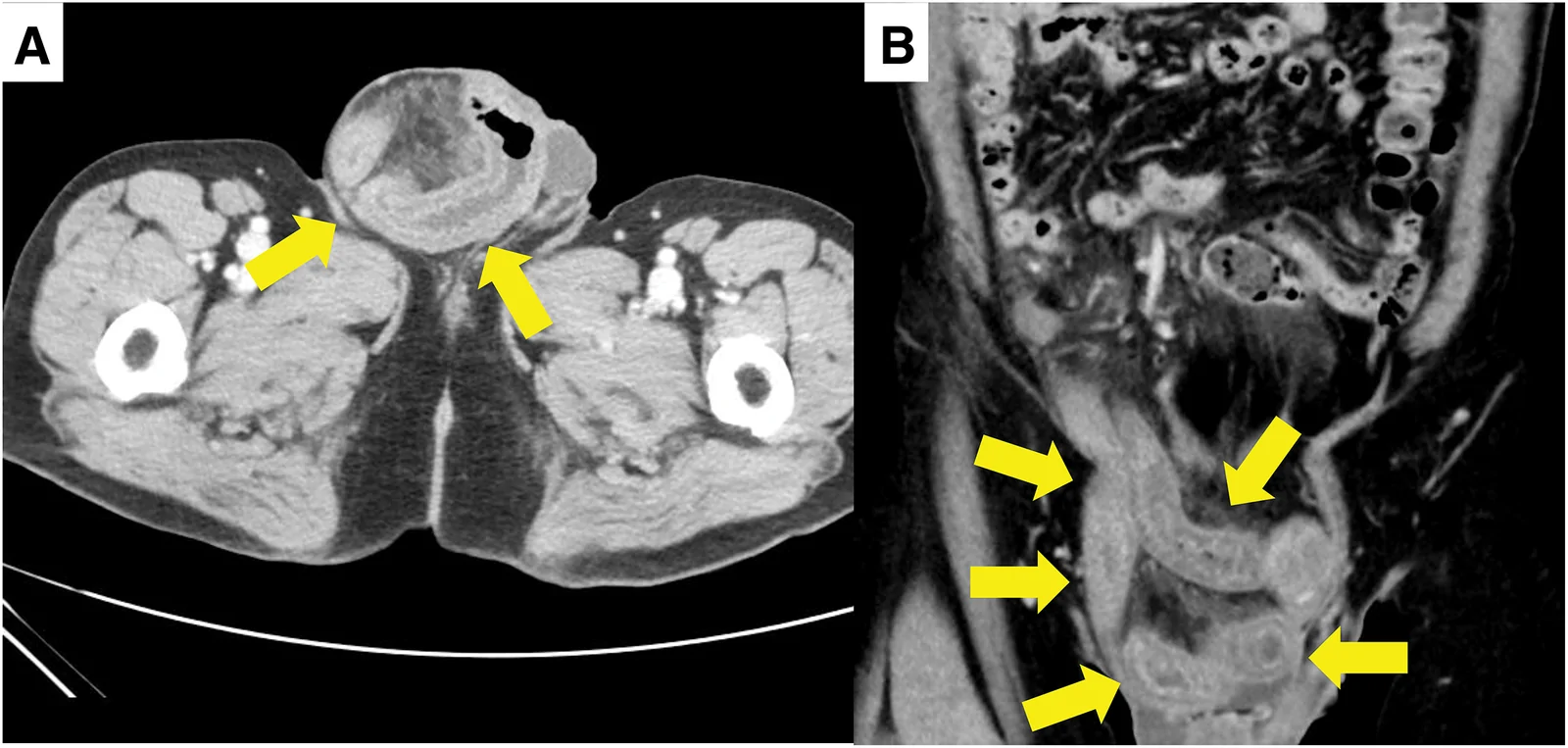
Preoperative contrast-enhanced CT findings. (**A**) Axial and (**B**) coronal images show a right inguinal hernia containing small bowel. The herniated bowel demonstrates wall thickening, surrounding mesenteric fat stranding, mildly reduced but preserved enhancement, and a small amount of adjacent ascites, suggesting possible impaired perfusion. Yellow arrows indicate the incarcerated bowel.

Because the hernia had been reduced, the abdominal symptoms improved, and there were no definitive signs of peritonitis or bowel necrosis, immediate emergency surgery was not performed. The patient was admitted for close inpatient observation, including repeated assessment of vital signs, abdominal symptoms, and peritoneal signs, with a plan for early same-admission repair on hospital day 4 if his condition remained stable. On the following morning, the white blood cell count had decreased from 13900 to 11600/μL. Although the C-reactive protein level increased from 0.23 to 2.07 mg/dL, the patient remained hemodynamically stable without peritoneal irritation or worsening abdominal symptoms. No clinical deterioration was observed during the observation period.

Elective laparoscopic TAPP was performed under general anesthesia on hospital day 4. At the start of surgery, the hernia contents had already been reduced. A camera port was inserted through the umbilicus using the open method, and two 5-mm ports were placed in the bilateral lower abdomen. Intraperitoneal exploration revealed a segment of ileum with reddish discoloration over several tens of centimeters in length. However, bowel peristalsis was preserved, and no obvious necrosis or perforation was identified (**Fig. 2A**–**2C**). The hernia was diagnosed as a right indirect inguinal hernia, classified as L3 according to the 2021 Japanese Hernia Society classification system. No left inguinal hernia was identified. The right hernia orifice was confirmed after reduction (**Fig. 2D**).

**Fig. 2:**
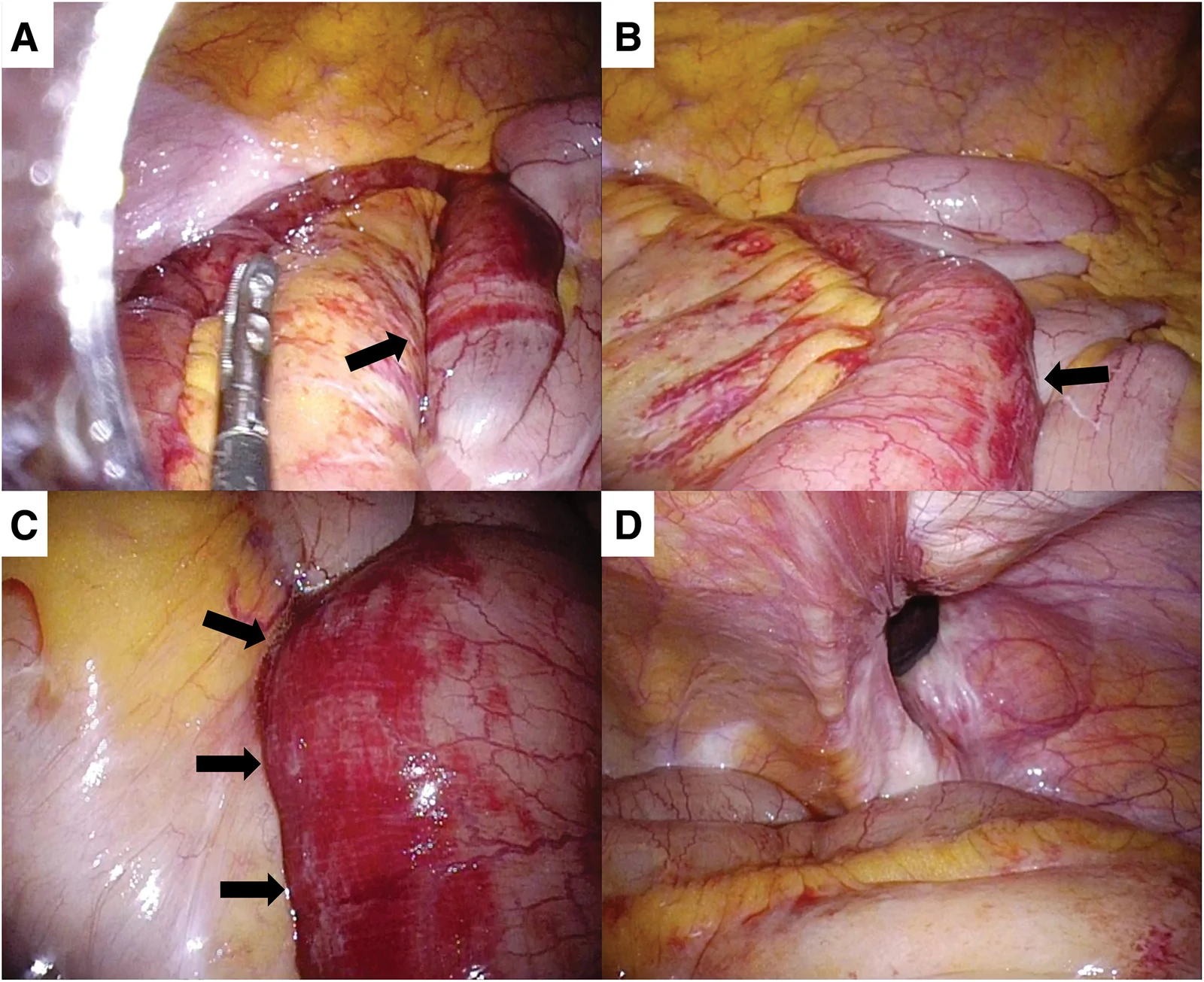
Intraoperative laparoscopic findings. (**A**) Overview of the previously incarcerated ileal segment after reduction. (**B**) The transition between the affected and macroscopically normal bowel. (**C**) Close-up view showing reddish discoloration of the affected segment; peristalsis was preserved, and no necrosis or perforation was identified. (**D**) The right indirect inguinal hernia defect after reduction. Black arrows indicate the affected ileal segment (**A**, **C**) and the transition between the affected and macroscopically normal bowel (**B**).

The peritoneum was incised lateral to the inferior epigastric vessels, and preperitoneal dissection was performed. The prevesical space, vas deferens, and testicular vessels were identified and preserved. A large 3D mesh was placed to cover the hernia defect with adequate overlap. The mesh was fixed to the rectus abdominis muscle, Cooper’s ligament, the area around the hernia defect, and the abdominal wall, while avoiding injury to the inferior epigastric vessels. The peritoneum was closed with continuous absorbable sutures. No bowel injury or bleeding was observed. The operative time was 80 min, anesthesia time was 120 min, and blood loss was minimal.

The postoperative course was uneventful. The patient had no signs of bowel obstruction, delayed perforation, mesh infection, or recurrent incarceration and was discharged without major complications. At 3 months after surgery, no recurrence, bowel obstruction, mesh-related complication, or chronic groin pain was observed.

## DISCUSSION

This case highlights 2 clinically important points. First, even when CT suggests possible impaired bowel perfusion in an incarcerated inguinal hernia, successful manual reduction followed by strict inpatient observation may allow same-admission elective repair in carefully selected patients. Second, laparoscopic TAPP can be useful after reduction because it allows direct assessment of bowel viability while providing definitive hernia repair.

The most important issue after successful manual reduction is to distinguish patients who can be observed safely from those who require immediate emergency surgery. Emergency surgery remains mandatory in patients with suspected bowel necrosis, perforation, peritonitis, persistent bowel obstruction, or ongoing strangulation, or in those with hemodynamic instability, metabolic acidosis, elevated serum lactate, or progressive abdominal symptoms.^2,5)^ Emergency surgery should also be selected when imaging suggests irreversible ischemia, perforation, pneumatosis intestinalis, portal venous gas, or closed-loop obstruction. Therefore, technical success of manual reduction alone should never be used as the sole reason to defer emergency surgery.

In the present case, CT showed bowel wall thickening, mesenteric fat stranding, slightly decreased enhancement, and ascites, all of which raised concern for strangulation. Nevertheless, delayed repair was not selected solely because manual reduction had been successful. The decision was based on several clinical factors: the interval from onset of incarceration to successful manual reduction was approximately 5 h, the patient was hemodynamically stable, symptoms improved after reduction, and there were no clear clinical signs of peritonitis. In addition, no definite evidence of bowel necrosis or perforation was identified. Laboratory findings showed leukocytosis and a mildly elevated lactate dehydrogenase level, but creatine kinase was not elevated. These factors supported close inpatient observation rather than immediate emergency bowel exploration.

The updated HerniaSurge guidelines suggest that manual reduction may be attempted in acutely irreducible groin hernias when bowel ischemia is not suspected, followed by observation after successful reduction.^2)^ Pawlak et al. proposed an algorithm for manual reduction using the GPS concept, emphasizing gentle reduction, preparation, and safety, and recommended its use only in selected patients without signs of strangulation.^3)^ East et al. also described manual reduction as a useful initial strategy in acute inguinal hernia, while emphasizing that emergency surgery remains the mainstay when strangulation is suspected.^4)^ The present case should not be interpreted as supporting routine manual reduction in patients with suspected bowel ischemia. Rather, it represents a carefully selected case in which reduction had already been achieved promptly, symptoms improved, and no definitive evidence of irreversible ischemia or peritonitis was present. Because preoperative CT suggested possible bowel ischemia, we considered inpatient observation and same-admission repair more appropriate than discharge after reduction.

Manual reduction is not risk-free. Reduction en masse, persistent closed-loop obstruction, delayed bowel ischemia, and perforation are recognized concerns after apparently successful reduction.^3,4,10)^ Therefore, careful observation is essential, particularly when preoperative imaging suggests bowel compromise. In the present case, observation was performed in the inpatient setting, and elective repair was performed on hospital day 4. This interval allowed repeated clinical assessment for delayed ischemia while avoiding prolonged postponement after the episode of incarceration.

The timing of definitive repair after successful reduction should be individualized. Immediate emergency surgery is appropriate when symptoms persist or worsen after reduction or when irreversible ischemia cannot be excluded. Conversely, outpatient elective repair after reduction may be reasonable in patients with low-risk findings and complete symptom resolution. The present case occupied an intermediate position: CT suggested possible bowel ischemia, but the clinical course after reduction remained stable. For this reason, inpatient observation followed by same-admission elective repair was considered appropriate. A concise proposed framework for management after manual reduction is summarized in **Table 2**.

**Table 2:** Proposed decision-making framework after successful manual reduction of an acutely irreducible groin hernia^2–5,11–13)^

Clinical findings after reduction	Suggested management
Hemodynamic instability; peritonitis; persistent or worsening abdominal pain; persistent bowel obstruction; or imaging findings suggesting irreversible ischemia, perforation, pneumatosis intestinalis, portal venous gas, or a persistent closed-loop obstruction	Immediate emergency exploration
Stable vital signs, complete or marked symptom improvement, no peritoneal irritation, and no definite evidence of irreversible bowel ischemia	Short observation followed by early elective repair, either during the same admission or after discharge
Clinical stability with equivocal risk, such as possible ischemia on imaging, recurrent groin bulging, or uncertainty regarding bowel viability	Inpatient observation with repeated clinical assessment and same-admission laparoscopic exploration and repair
Viable bowel without perforation or gross contamination at laparoscopy	Mesh repair may be performed
Bowel necrosis, perforation, or gross contamination at laparoscopy	Bowel resection as required; non-mesh tissue repair or staged mesh repair according to contamination and patient factors

This framework is proposed for clinical decision-making and has not been validated as a standardized protocol.

Evidence directly comparing emergency surgery with elective repair after successful manual reduction remains limited. Kohga et al. retrospectively analyzed 112 patients with incarcerated groin hernias: 76 underwent emergency surgery and 36 underwent elective surgery after reduction. Among patients who did not require bowel resection, the reduction group had a shorter postoperative hospital stay (4.3 vs. 8.0 days), more frequent mesh use (100% vs. 74.4%), and fewer postoperative complications (13.8% vs. 45.4%).^11)^ Bouliaris et al. prospectively reported successful taxis in 57 of 86 patients with incarcerated abdominal wall hernias; 52 patients with successful reduction and substantial pain improvement completed 24-h inpatient observation and were subsequently scheduled for elective repair.^12)^ Although these findings support elective repair after successful reduction in selected patients, direct application to the present case requires caution because the study populations were heterogeneous and patients with more severe strangulation were more likely to undergo emergency surgery.

The laparoscopic transabdominal preperitoneal approach was particularly useful in this case. In an open anterior repair, the previously incarcerated bowel may not be inspected directly unless an additional abdominal incision is made. By contrast, TAPP permits intraperitoneal assessment of the bowel before mesh placement. Previous studies and case series have reported that laparoscopic transabdominal repair is feasible for selected incarcerated or strangulated inguinal hernias and allows assessment of hernia contents and bowel viability.^6–9)^ A recent systematic review of 8 studies involving 316 patients undergoing emergency TAPP reported 3 conversions to open surgery, 2 recurrences, 25 visceral resections, and no deaths.^13)^ Although these studies primarily involved emergency repair rather than same-admission elective repair after manual reduction, they support the feasibility of TAPP and its value for direct intraperitoneal assessment in selected patients. In the present case, laparoscopic exploration demonstrated reddish discoloration of the ileum, but bowel peristalsis was preserved, and there was no necrosis, perforation, or gross contamination. The decision to avoid bowel resection was based not only on preserved peristalsis but also on the absence of irreversible ischemic changes, perforation, and contamination.

Mesh use in the setting of incarceration requires careful consideration. International groin hernia guidelines recommend mesh repair as a standard approach for groin hernias.^1,2)^ WSES guidelines for complicated abdominal wall hernias also emphasize that the decision to use mesh in emergency settings should consider the presence or absence of contamination, bowel resection, and infection risk^5)^. In the present case, there was no bowel resection, perforation, or contamination, and therefore mesh repair was considered acceptable. A 3D mesh was placed using the transabdominal preperitoneal approach, and no mesh-related complication was observed during the 3-month follow-up period.

If bowel necrosis, perforation, or gross contamination had been identified during laparoscopic exploration, mesh placement would have been abandoned. Bowel resection would have been performed through a small laparotomy or laparotomy as required, followed by non-mesh tissue repair or staged mesh repair after resolution of contamination and inflammation. If bowel resection had been required without perforation or gross contamination, the decision regarding mesh use would have been individualized according to bowel viability, the degree of contamination, and the patient’s condition. Thus, laparoscopic exploration was useful not only for confirming bowel viability but also for determining whether mesh repair was appropriate.

This case has several limitations. It is a single case, and the findings should not be generalized to all patients with incarcerated inguinal hernia. Our institutional experience with this strategy remains limited, and we have neither a standardized institutional protocol nor a comparative institutional cohort for same-admission elective laparoscopic repair after manual reduction. Therefore, management was individualized on the basis of the patient’s clinical course and the available literature. The decision to observe after manual reduction should be limited to carefully selected patients with stable vital signs, improvement of symptoms after reduction, absence of peritoneal irritation, and no definite evidence of irreversible bowel ischemia. The current follow-up period is relatively short. Continued clinical follow-up is planned at 6 and 12 months and annually thereafter to assess late recurrence, chronic postoperative inguinal pain, bowel obstruction, and mesh-related complications.

## CONCLUSIONS

Successful manual reduction followed by strict inpatient observation allowed same-admission elective laparoscopic TAPP in a patient with incarcerated inguinal hernia and CT findings suggestive of possible small-bowel ischemia. Laparoscopic repair was useful because it enabled both direct assessment of bowel viability and definitive mesh repair. This strategy may be an option in carefully selected patients, but delayed ischemia, persistent obstruction, and reduction en masse must always be considered after manual reduction. The decision to defer emergency surgery should be made cautiously and should not be based on the technical success of reduction alone.

## DECLARATIONS

### Availability of data and materials

The datasets used during the current report are available from the corresponding author on reasonable request.
